# Western Virginia Medical Society

**Published:** 1872-06

**Authors:** 


WESTERN VIRGINIA STATE MEDICAL SOCIETY. The Medical Society of West Virgina held their session in Wheeling on the 5th, 6th and 7th of June. From what we can gather from the brief reports which have come under our notice, the session was a profitable and interesting one. The difficulty with the late secretary Dr. James Reeves was adjusted to the satisfaction of all by a letter of apology from that gentleman which explained some things which had not before been understood. After listening to an able valedictory address by the president, Dr. Lazzell, the society adjourned to meet at Parkersburg, Mineral Wells on the first Wednesday in June 1873.

